# Comparison of Human Primary with Human iPS Cell-Derived Dopaminergic Neuron Grafts in the Rat Model for Parkinson’s Disease

**DOI:** 10.1007/s12015-015-9623-7

**Published:** 2015-10-05

**Authors:** Su-Ping Peng, Sjef Copray

**Affiliations:** Center for Neuroscience, Shantou University Medical College, Shantou, Guangdong Province People’s Republic of China; Department of Neuroscience, Medical Physiology, University Medical Center Groningen, University of Groningen, A. Deusinglaan 1, 9713 AV Groningen, The Netherlands

**Keywords:** iPS cells, Ventral mesencephalic dopaminergic neurons, Foetal tissue, Neurite extension and Parkinson’s disease

## Abstract

Neuronal degeneration within the substantia nigra and the loss of the dopaminergic nigro-striatal pathway are the major hallmarks of Parkinson’s disease (PD). Grafts of foetal ventral mesencephalic (VM) dopaminergic (DA) neurons into the striatum have been shown to be able to restore striatal dopamine levels and to improve overall PD symptoms. However, human foetus-derived cell grafts are not feasible for clinical application. Autologous induced pluripotent stem cell (iPS cell)-derived DA neurons are emerging as an unprecedented alternative. In this review, we summarize and compare the efficacy of human iPS cell-derived DA neuron grafts to restore normal behaviour in a rat model for PD with that of human foetal primary DA neurons. The differences we observed in the efficacy to restore normal function between the 2 types of DA neuron grafts could be ascribed to intrinsic properties of the iPS cell-derived DA neurons that critically affected survival and proper neurite extension in the striatum after implantation.

## Introduction

Parkinson’s disease (PD) is one of the most prevalent neurodegenerative diseases, affecting 1 in 100 people over the age of 60 [1]. The disturbances in the initiation and fine-regulation of movement resulting in tremor, rigidity, akinesia and postural instability in PD patients are caused by the progressive loss of dopaminergic (DA) neurons in the substantia nigra (SN). The loss of DA neurons in the SN leads to a disruption of the nigro-striatal circuitry and a dopaminergic depletion of the striatum. The current standard therapy, L-DOPA administration, only temporarily restores striatal dopamine levels whereas deep brain stimulation only transiently reduces severe tremor [2, 3].

The search for more efficient and long-term effective treatment strategies for PD is still ongoing. The ground-breaking developments in stem cell research in the last decade have revived the interest for intracerebral cell transplantation as a therapeutical approach for PD. In this approach, replacement of the lost nigrostriatal dopaminergic innervation of the striatum by exogenous dopaminergic neurons is intended to restore basic dopamine levels within the striatum. Experiments on rodent and non-human primate models for PD in the 1970s and 1980s, had shown that embryonic DA neurons could survive and reinnervate the striatum after stereotactic injection [4, 5]; moreover, reduction in drug-induced rotation behaviour in’Parkinsonian’ rats demonstrated that the grafted DA neurons indeed functionally integrated into the striatum and released dopamine [6–8]. Based on the findings in the experimental animal models, various clinical trials were started in the 1980s and 1990s with the striatal implantation of human foetal DA neurons with promising results. Patients with human foetal ventral mesencephalon (VM) grafts have been shown to recover from rigidity and tremors to various degrees, correlated to the restoration of intracerebral dopamine levels as detected by PET [9–11]; many of these patients experienced an overall improvement in quality of life [9, 10, 12, 13]. Though the outcome of striatal implantation of human foetal DA neurons in a large group of PD patients was very promising, reports on severe side effects, presumably due to suboptimal, poorly documented graft composition and/or adverse implant localization, led to a discontinuance of this approach. Apart from the complicated logistics to obtain multiple donor foetuses of the same developmental stage, the use of abortion-derived human foetal brain tissue to obtain a sufficient number of homogenous graftable DA neurons raised considerable ethical concern.

Several other sources for implantable human DA neurons have been explored ever since. DA neurons have been successfully differentiated in-vitro from human embryonic stem (ES) cells and human neural stem cells (NSCs). Besides the fact that both sources still caused ethical objections, the non-autologous origin of these cells, demanding lifelong immunosuppression after implantation, was considered a major obstacle. The ground-breaking detection of induced pluripotent stem cells (iPS cells) generated from easily accessible somatic cells (e.g. skin fibroblasts) [14, 15] has provided an unprecedented novel autologous source for human DA neuron grafts. The functionality of these in-vitro generated human DA neurons after intrastriatal implantation have been recently studied in rodent and non-human primate models for Parkinson’s disease: in general, apart from the assessment of motor behaviour, parameters such as neuronal survival, the extent of neurite outgrowth, the coverage of the striatum with a fine network of dopaminergic terminals and the release of dopamine have been evaluated.

The aim of this review is to summarize the behavioural effects, the survival and neurite outgrowth of human iPS cell-derived DA neurons after their intrastriatal implantation in the Parkinson rat model and compare them with those observed for implanted human primary foetal DA neurons. We discuss possible explanations for the differences in efficacy, survival and outgrowth between the iPS cell-derived DA neuron grafts and the primary DA neuron grafts.

## Unilateral 6-OHDA Lesion Rat Model for Parkinson’s Disease

In order to study the feasibility and efficacy of grafted DA neurons as therapy for Parkinson’s disease, a proper animal model is required. Among various animal models for PD (for review see [16, 17]), 6-OHDA-lesioned rats are the most often used PD animal model in DA neuron graft research. The toxin 6-hydroxy-dopamine (6-OHDA) specifically uses the catecholamine transport system of catecholaminergic neurons to enter the cells. It generates hydrogen peroxide and hydroxyl radicals and disturbs mitochondrial complex I, which leads to the production of superoxide free radicals and eventually to cell death. Stereotactical injection of 6-OHDA into the striatum of an adult rat, will induce retrograde degeneration of the SN DA neurons, usually within 2 to 3 weeks [18]. When the injection of 6-OHDA is directed into the nigrostriatal tract or in the SN itself, acute DA neuron death will be induced within 24 h [19]. The size of the lesion can be adjusted by varying the dosage of 6-OHDA [18]. The efficacy of the unilateral 6-OHDA induced lesion and so the effect of a specific treatment in rats are validated by ipsilateral or contralateral circling motor behaviour after the administration of amphetamine or apomorphine [20]. The number of rotations per minute can be taken as a measure for the severity of unilateral DA loss in the striatum and the efficacy of compensation by DA-producing grafted DA neurons [21]. Besides drug-induced rotation, 6-OHDA unilateral lesions of the rodent meso-telencephalic dopamine pathways also lead to postural curvature, spontaneous rotation, contralateral sensory neglect and aberrant activity [7, 22]. Various activity tests, such as the adjusting step test [23] and the cylinder test [24], have been used to study the non-rotational behavioural consequence of the 6-OHDA unilateral lesions and the effects of a specific treatment on that.

## Human Foetal Dopaminergic Neuronal Grafts

Since the late 1970s, transplantation of embryonic VM tissue from rodents and primates in PD animal models has been extensively studied (for reviews see [25, 26]). From these fundamental studies, protocols for transplantation were established as paradigm for studies on human foetal tissue transplantation. It was established that only newly formed postmitotic DA neurons (E13 - E15 in rats) are suitable for grafting, since they have just reached a dopaminergic phenotype but do not yet have fully outgrown extensions, which makes it possible to isolate them without too much cellular damage [21, 27]. Extrapolating these data to human DA neuron grafts, human foetal VM tissue of a gestational age of 7 to 10 weeks was considered optimal. In almost all rodent studies, transplantation was performed right after tissue isolation and preparation, either dissociated into cell suspension or dissected as small tissue pieces. In this approach, DA neurons appeared to be still viable and survive the grafting procedure [26, 28].

Based on the promising data of rodent and primate DA neuron-rich VM tissue transplanted into PD models [4, 8, 21, 29–31], experimental studies with the implantation of human foetal DA neurons in PD models were started.

### Cellular Composition

When isolating and dissociating the foetal human ‘DA’ VM region for Parkinson-related transplantation purposes, one should be aware of the actual cellular composition of this region. This is of considerable importance since in most PD transplantation studies foetal human VM cell suspensions have been used without any form of purification. In the human (foetal) VM region, SN DA neurons and ventral tegmentum area (VTA) DA neurons indeed make up around 70 % of the neurons [32]. About 30 % of the neurons are GABAergic; in addition 2–3 % of glutamatergic (Glu) neurons can be found in this region [32]. However, one should be aware that neurons account for only 5.6 % of the total number of cells in the VM, which is even less than in most other brain areas, for instance the brain stem; 8 % of the cells in this area are neurons [33]. Most of the cells in the VM region are nonneuronal and comprise astrocytes, microglia, endothelial cells etc.… The contamination of all these specific nonneuronal cell types in the ultimate graft will inevitably affect the survival and function of the implanted DA neurons and the reaction of the host in the environment around the graft. So, in fact, only a minority of cells in the non-purified foetal cell suspension from the human VM region, isolated for grafting in PD animal models, can be actually considered SN dopaminergic neurons. When human VM tissue grafts are collected from aborted foetuses, the cellular composition may be even more uncertain, due to the method of abortion used. In many cases, the abortion procedures led to a destruction of the foetus, which made it difficult to recognize brain tissue, let alone the very small specific VM portion. Even contaminating serotonergic (!) neurons have been demonstrated in the periphery of the grafts in the study by Stromberg in 1989 [34]: many 5-HT-immunoreactive terminals were found in the striatal neuropil of the host brain arising from the graft. Most studies do not even report or discuss the cell composition of the used human foetal DA neuron grafts.

### Efficacy of Intrastriatally Grafted Human Foetal DA Neurons in PD Animal Models

#### Behavioural Improvement and DA Neuron Survival

In most studies on human foetal VM tissue transplantation in the unilaterally 6-OHDA lesion rat PD model (Table 1), a significant reduction in drug-induced rotation was observed either after grafting with a VM-derived cell suspension or small VM tissue pieces. In two rats, in the study by Brundin et al., rotation was completely annihilated and even contralateral rotation was induced at 12.5 weeks post grafting. Another two rats in this study reduced amphetamine-induced rotation by 69 and 92 % at week 15.5 post grafting [35]. Rotation reduction appeared to be correlated with the number of surviving DA neurons: complete recovery was established by an average of 1200 DA neurons 21 weeks post grafting, while the 14 to 107 DA neurons found in some rats were unable to induce any behavioural change [35]. The behavioural improvement after human DA grafting occurred much later than what was observed with rat embryonic VM tissue grafts (3–4 weeks post grafting), indicating that human neurons required more time to mature and integrate into the striatum. Reduction in drug-induced rotation was only observed 15.5 weeks post grafting in rats receiving a human VM cell suspension from foetuses of gestational age of 9 weeks (PC 9 weeks), but not from foetuses isolated at gestational age between PC 11–19 weeks [35]. In another experiment with a similar set-up, intrastriatal grafting of human VM tissue with a gestational age of 6.5 weeks and 8 weeks resulted in a pronounced reduction in both amphetamine- and apomorphine-induced rotation, as well as in spontaneous rotation 18 weeks post grafting [37]. In these animals, 300 to 4249 (average 1626) DA neurons were found to survive in the grafted striatum. The studies by Brundin et al. indicated that in general between 1 and 10 % of the grafted human foetal VM cell suspension (approximately 40,000 DA neurons) survived the implantation procedure [35, 37]. Additional studies have shown that the survival of between 500 and 700 human foetal VM DA neurons are needed for full reversal of the amphetamine-induced rotation behaviour by 19–21 weeks post grafting [38, 44].

**Table 1:** Summary of grafting of human VM tissues into rat PD model

[Ref],Donor age, (mode)/graft site	Functional recovery reduction of rotation	Tissue survival (number of DA neurons)	Characteristics of grafted DA neuron	Neurite outgrowth & striatum reinnervation (%)
[35], PC 9-16w, (cells); PC 16-19w, (pieces)/CPu	**Amphetamine:** PC 9w: 69–92 % (15.5 WPG) >100 % (20 WPG) PC11-19w: <0 %	PC-9w: 300–1800 (10, 21 WPG) PC-11w: 14–74 (6, 12.5 WPG) PC15-19w: 0–2 (5, 20 WPG)	**Morphology:** **1.** Bipolar appearance **2.** Long coarse processes **3.** Diameter of cell body 50 μm	Extended 2–3 mm, dense close to graft site; sparse further away
[36], PC 9-12w, (pieces)/CPu	**Apomorphine:** 50–80 % (4–5 MPG)	Moderate to large numbers	**Morphology:** **1.** 2–3 MPG: small rounded cell bodies **2.** 4 MPG: similar to adult SN neurons	40-60 % of the dorsal striatum Fibers also reaching ventral parts
[37], PC 6.5-11.5w, (cells)/CPu	**Amphetamine:** PC 6.5-8w: > 94 % in 6 out of 7 rats (18 WPG) PC 11.5w: 13–52 % in 4 rats (19.5 WPG) **Apomorphine:** PC 8w: 58–79 % (18 WPG) **Spontaneous rotation:** >100 %	PC 6.5-8w: 402–4249 PC-11.5w: 0–198 No surviving of DA neurons without immunosuppression	**lntracerebral dialysis:** **1.** Basal extracellular DA levels (intact striatum): 0.26 ± 0.08 (0.19 ± 0.06) pmol/25 μl **2.** Activated DA levels: Amphetamine, 3.7 (15.12) folds; nomifensine, 3 (5.79) folds **Morphology:** Diameter of cell body: 50 μm; mean 25 × 19.5 μm; coarse TH+ processes **Migration:** 1–1.5 mm	Extensive network reinnervated 100 % of the head of the caudate-putamen Extending up to at least 6 mm
[38], PC 6.5-9w, (cells)/CPu	**Apomorphine-induced rotation:** >100 % (19–21 WPG)	500–700	**Morphology:** Diameter of cell body: 50 μm; mean 12 × 16 μm Multipolar and of irregular shape Coarse TH+ dendrites **Synapse formation:** 8 WPG: TH- synapse 11 WPG: some TH+ synapses 20 WPG: TH+ synapses throughout the neostriatum TH+ boutons 0.6–1.2 μm in diameter	8 WPG: no fiber outgrowth 11 WPG: sparse TH+ fiber 20 WPG: rich fiber network Reinnervated 100 % of neostriatum
[39], PC 7-12w, (pieces)/CPu	n.d.	n.d.	**Electrophysiology (locally applied K+):** 50 % amplitudes of electrochemical signals of normal control; 2 folds of denervated area **Morphology**: TH+ processes contained vesicles; Formed symmetric and asymmetric synapses with host dendritic spines and shafts	TH+ neurites extended a few millimeters from the graft
[34], PC 7-12w (pieces)/CPu	n.d.	1. Large amount of TH+ neurons 2. Numerous 5-HT+ neurons	**Morphology**: **1.** Oval or rounded cell body, diameters 40 μm **2.** >1 primary dendrites **3.** Thick TH+ fibers **4.** Vesicle-filled axons made close symmetric/ asymmetrical synapses with dendritic shafts and dendritic spines **Electrophysiology (locally applied K+):** Signals amplitude of 0.5 ± 0.1 mM (for normal control 2.0 ± 0.1 mM)	1. TH+ fibers extended to 2.5–3 mm 2. Innervations of 5-HT fibers
[40], PC 7-12w (pieces)/CPu	78 % (5 MPG)	1. TH+ cells were found 2. Few 5-HT+ cells	**Morphology**: Oval, fusiform or triangular cell body Thick TH+ fibers **Electrophysiology:** **1.** Action potentials: bi- or triphasic, >1.5 msec durations (similar to normal DA neurons) **2.** Patterns of spike activity: ‘activate’ in 14/41 cells recorded; ‘partially active’ in 27/41 cells recorded **3.** Slow discharge rate (2.9 ± 0.4 spike/s) (normal control 3.0 ± 0.4 spike/s)	TH+ neurites reinnervated 100 % of the striatum No increase in 5-HT-positive fibers
[41], PC 6-8w, (cells)/Rostral mes; IC; CPu	n.d.	13–20 WPG, low number	n.d.	**Rostral mes & IC:** Neurites (majority TH+) extended along the medial forebrain bundle, even up to the olfactory bulb (10 mm); neurites ramified within the caudate putamen **CPu:** Most neurites ramified and terminated within the caudal caudate putamen, a few extended caudally
[42], first trimester fetus, (pieces)/ lateral ventricle	**Apomorphine:** Completely eliminated in 3 rats; 93 % decrease in 1 rat (7 MPG)	n.d.	**Striatal response to graft:** Reversed the increased of D2 receptors The decrease of D1 receptors remained Normalized the increased of D3 receptor	hNF+ cover 100 % of striatum
[43], first trimester fetus, (pieces)/lateral ventricle	**Apomorphine:** 86 % (4MPG)	Numerous TH+ neurons	**MPTP response:** TH+ reinnervated fibers in dorsal striatum were degenerated TH+ reinnervated fibers in olfactory tubercle were spared	TH+ fiber densely innervated 100 % of striatum
[44], PC 7-9w, (cells or pieces)/CPu or SN	**Apomorphine:** Part/metal/CPu = 75 % ≈ Full/glass/CPu ≈ Full/glass/SN > Full/metal/ CPu > Sham/glass/SN = Sham/metal/CPu **Amphetamine:** Part/metal/CPu > 100 > Full/glass/CPu = Full/metal/ CPu > Full/glass/SN = Sham/glass/SN = Sham/metal/CPu	Pieces/metal/CPu: 2183 ± 449 Cells/metal/CPu: 892 ± 378 Cells /glass/CPu: 657 ± 199 Cells/glass/SN: 1548 ± 362	VMAT+/TH+ co-labeling	Cells/metal/CPu: Reinnervation throughout the entire striatum, extended up to caudal brain regions (5–9 mm) Pieces/glass/SN: hNF70+ fibers exclusively confined to the SN and the needle tract

*Abbreviations: PC* Post-conception, *CPu* Caudate-putamen, *SN* Substantia nigra, *mes* Mesencephalon, *IC* Internal capsule, *cells* Single cell suspension, *Metal* Metal cannula, *Pieces* Tissue pieces, *glass* Glass capillary, *WPG* Weeks post grafting, *MPG* Months post grafting, *n.d.* not determined/not described

Transplantation of small VM tissue pieces in a premade cavity in the cortex also yielded significant DA neuron survival and led to a significant reduction of drug-induced rotation. In this approach, even human VM tissue pieces of a gestational age up to 12 weeks could actually survive transplantation and gave rise to a similar behavioural improvement at 3–5 months post grafting as the cell suspension grafts [34, 36]. The generation of human VM DA neurons takes place during post conception week 6.5 to 9; they start extending elaborated processes towards the striatum from week 10 on [45]. It is apparent that the DA neurons in young explants (PC 6.5–9 weeks), since they do not have extensive axonal and dendritic outgrowth, are hardly damaged during isolation and dissociation; in contrast, older foetal DA neurons (PC 11–19 weeks) already have extensive axons, dendrites and connections, so most of them are severely damaged after dissociation and will not survive after implantation. When dissociation is omitted and small blocks of foetal VM tissue are used, the structure of most cells will be preserved, so that even tissues from PC 12 weeks can be used.

Full reversal of apomorphine-induced rotation was seldom reported. Yet, Stromberg et al. showed that this is possible with prolonged time, 7 months post grafting. The increase of D2 and D3 receptors in the striatum seems to be normalized at this time point [42]. It has been suggested that the function of SN also depends on dendritic DA release in the SN [46]. Interestingly, transplantation of DA neurons in the SN can give rise to a reduction of apomorphine-induced rotation [44]. However, it is still controversial whether the DA neurons grafted close to SN are able to extend through the meso-striatal pathway to reinnervate the striatum; whereas Rath et al. [44] were unable to detect such connection, both Wictorin et al. [41] and Grealish et al.[47] showed that grafts of human foetal VM of different donor ages could indeed reconstruct the meso-striatal pathway when grafted to SN of adult 6-OHDA lesioned rats.

#### Characteristics of Transplanted Human Foetal DA Neurons

It was observed that human foetal DA neurons grafted in the striatum needed a longer period to mature and integrate than those from rodents. Two to three months post grafting, the human TH+ neurons still had an immature small rounded cell body. Up to 4 months, the DA neurons were more similar to adult SN DA neurons with TH+ dendrites and often with fine spine-like lateral processes [36]. Two main populations of TH+ neurons were observed: the most frequent type with small perikarya of diameter between 12 and 25 μm, and the rest of the population with a large perikarya diameter up to 50 μm long [37, 38]. In some cases, TH+ perikarya were found up to 1–1.5 mm from the graft borders in the host striatum, indicating the ability of the grafted premature DA neuroblasts to migrate [37]. Most of the neurons were multipolar with coarse processes extending 2.5–3.0 mm [34] into the host striatum, and in some case, up to 6 mm [37] or even 9–10 mm [41, 44]. The extent of actual reinnervation of the striatum correlated to the post grafting time. A sparse TH+ fibre plexus was seen adjacent to the implant 11 weeks post grafting, while a rich strong TH+ fibre network appeared to have reinnervated the entire striatum at 20 weeks (or longer) post grafting [38, 40, 42, 43].

The demonstration of reciprocal synaptic connectivity between grafted human foetal DA neurons and the host striatal interneurons confirmed the integration of the grafted human foetal DA neurons. Ultrastructural studies identified the TH+ coarse processes as dendrites [38], providing a site for host input to the graft: graft-derived TH+ dendrites appeared to receive non-TH labelled synaptic contacts in the host striatal neuropil. Moreover, TH+ axons containing round or oval vesicles formed symmetric synapses, like those seen in the normal meso-striatal DA pathway, with dendritic shafts and spines and in an even higher incidence on neuronal perikarya [34, 38, 39].

The electrophysiological characteristics of grafted human DA neurons were similar to those of DA neurons in an intact rat, which fire spontaneously in a slow firing rate (1–10 Hz) [48]. Some of the grafted human DA neurons showed the characteristic features of initially positively deflecting, bi- or triphasic action potentials with durations greater than 1.5 msec. The sustained activity rate of these DA neurons ranged between 0.1 and 10 spikes per second. As also found in the intact rat, some ‘partially active’ grafted human DA neurons exhibited action potential waveforms that were consistent with the waveforms recorded from in situ active DA neurons [40].

The functionality of the synapses formed by the grafted human DA neurons with the host striatal interneurons was validated with striatal recordings. The striatal interneurons at the site of the human foetal graft displayed slow discharge rates (3.1 ± 0.4 spikes/s) that were almost identical to those found in striatal neurons at the nonlesioned site (mean 3.0 ± 0.4 spikes/s) [40].

Intracerebral dialysis showed that the basal level of dopamine in the grafted striatum (0.26 ± 0.08 pmol per 25 μl of sample perfusate) of the 6-OHDA lesion rat was restored to the level observed in intact striatum (0.19 ± 0.06 pmol per 25 μl of sample perfusate). Grafted human DA neurons were able to respond to amphetamine stimulation with a significant increase in the DA level of the grafted striatum (3.7 times increase), although this increase was less pronounced than that observed in intact striatum (15.1 times increase) [37].

### Application of Human Foetal VM Tissue Transplantation in Parkinson Patients

The promising results of intracerebral implantation of human foetal VM DA neurons in the rat model for Parkinson’s disease led to open clinical trials in Parkinson patients. In these open clinical trials, many patients with human foetal VM grafts recovered from rigidity and tremors in various degrees, correlated to the restoration of the striatal dopamine level detected by PET [9–11]; the patients experienced an overall improvement in life quality [9, 10, 12, 13]. Occasional post mortem studies showed the survival of DA neurons at the graft sites (between 80,000 and 135,000 TH+ neurons), and extensive reinnervation of the striatum [49, 50]. The positive results of these initial open clinical trials sharply contrasted with the results of double-blind control NIH studies (reviewed by [51–53]): these studies failed to show any significant benefit in grafted patients up to 1 and 2 years in follow-up studies. Moreover, approximately 15 % of the grafted patients in NIH study I and 56 % in study II experienced severe graft-induced dyskinesias [54, 55]. The major differences in the outcome of graft treatment between the more accurate double-blind control NIH studies and the non-standardized open trials could be ascribed to major differences in the selection of patients, in the preparation, the storage, the final composition and the size of the human foetal cell graft, the site of intrastriatal deposition and the assessment methods. Although the impure composition of the human foetal grafts did not seem to prevent significant rotation reductions in the Ungerstedt rat model for Parkinson, it may be of major relevance for clinical application. Apparently, the complex architecture, composition, size and regional differences in functionality of the human striatum represent a completely different niche than the smaller, less complicated striatum of the rat. Recently, a number of post-mortem brain analyses were performed on Parkinson patients that received a foetal DA graft from 3 up to 16 years ago and more of such analyses are expected in the near future. Such analyses revealed the extent of survival and the cellular composition of the graft. The first data clearly showed that the grafted dopaminergic neurons were integrated in the striatal circuitry; in addition, interestingly, some of the grafted DA neurons appeared to have developed a Lewy body pathology pointing to a prion-like transfer from surrounding cells of the responsible pathogen, most likely alpha-synuclein [56–58].

It is clear that the development of autologous induced pluripotent stem cells has now provided an unrestricted source of graftable human DA neurons that can be extensively characterized and stringently purified before actual implantation in the Parkinson patients. Clinical application of these iPS cell-derived DA grafts in the near future can benefit from the ample practical experience with human foetal DA neuron intrastriatal implantations, despite their much-debated outcome. However, before clinical application, it needs to be established that DA neurons differentiated from human iPS cells show similar survival and neurite outgrowth after implantation in the PD animal models with a similar (or even a more pronounced) behavioural improvement as the primary foetal human DA neurons.

## Human iPS Cell-Derived Dopaminergic Neuron Grafts

Reprogramming of human fibroblasts towards iPS cells involves a complete epigenetic resetting and a concomitant complete cellular rejuvenation. Subsequent in-vitro differentiation and maturation of human iPS cells towards a dopaminergic cell lineage will yield foetal DA neurons, with a maturational stage comparable to the human DA neurons isolated from aborted foetuses. Proper DA differentiation largely depends on the completeness of the reprogramming process and on the in-vitro protocol used for differentiation. Such a protocol should meticulously recapitulate the normal embryonic development of DA neurons, mimicking the local micro-environment. Extensive characterization and purification should lead to a much better defined DA neuron cell suspension for implantation in comparison to the human foetal primary DA cell grafts.

### Cellular Composition of Human iPS Cell-Derived Dopaminergic Neuronal Graft

**Fig. 1:**
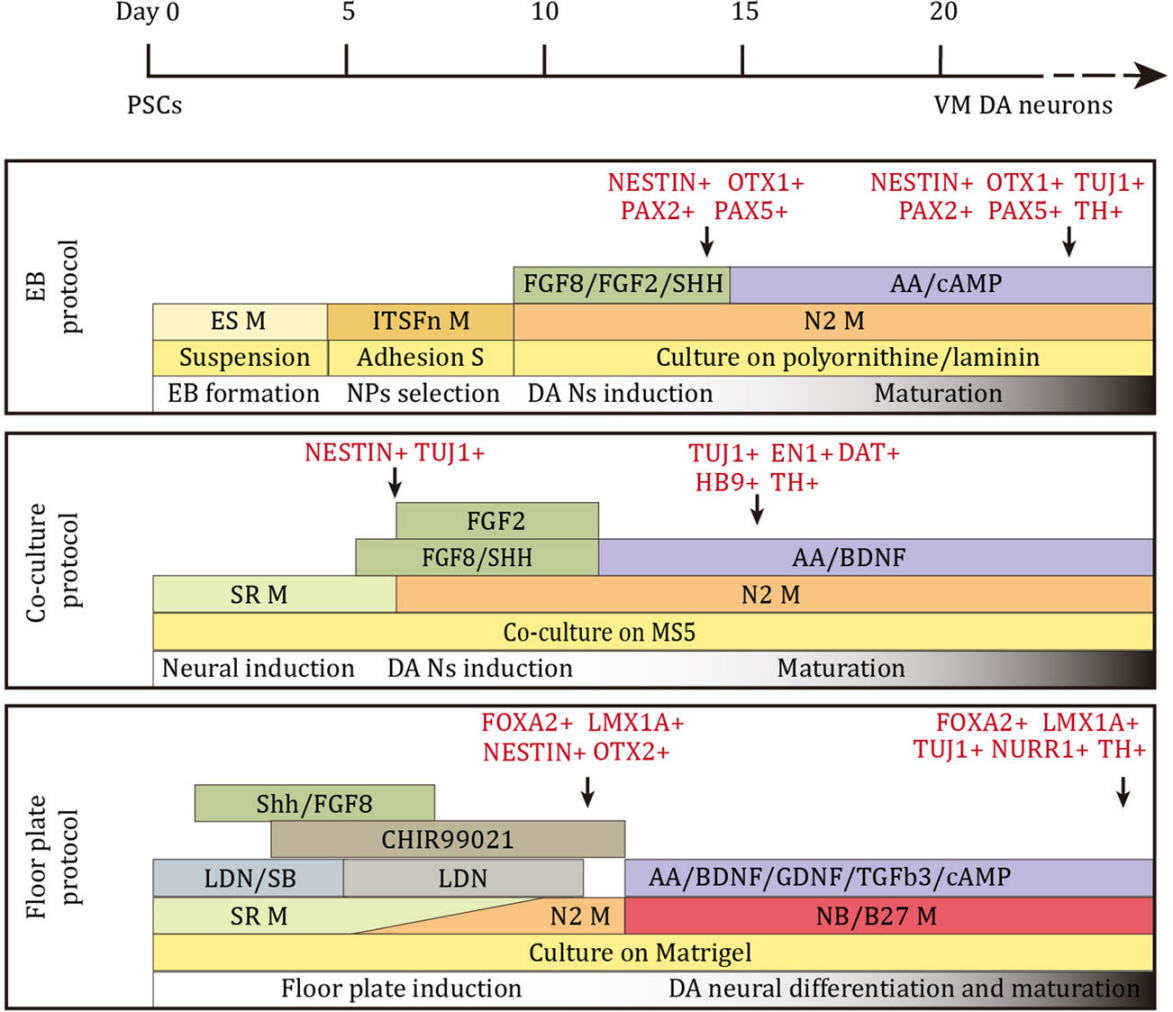
Schematic summary of the conditions for DA neuron differentiation in the representative protocols. Respectively for the EB method, the stromal cell co-culture method and the floor plate method. Based on [59] (EB), [60] (co-culture) and [61] (floor plate)

In order to generate proper VM DA neurons in-vitro from hiPS cells, three different differentiation strategies have been applied that have been developed for embryonic stem cells (ES Cells): the embryonic body (EB) method [59], the stromal cell co-culture method [60] and the defined-medium floor plate induced method [61] (for reviews see [62–66]). The representative procedure of each differentiation method and the expression of VM DA neuronal markers in differentiated cells are summarized in Fig. 1. Recently, several hiPS DA-differentiation protocols have been forwarded but in general they are just modifications of the 3 mentioned strategies [67–69].

The EB-based differentiation protocol starts with the spontaneous differentiation of cultured iPS cells into EBs containing the 3 germ lineages and is followed by the selective enrichment of the neuroectoderm and the neural precursors [59]. The stromal cell co-culture method is based on the stromal cell-derived inducing activity (SDIA) that has been shown to promote neural differentiation of ESCs [70]. In both methods, neural rosette structures are formed, which are composed of neuroepithelial cells [71, 72]. The defined-medium floor plate induced method employs dual inhibition of SMAD signalling by Noggin and SB431542 to force the differentiation of human ES Cells and iPS cells into a neural cell lineage [73]. Early exposure to SHH from day 1 of differentiation directs the iPS cells to a floor plate fate [74], and in combination with the WNT signalling activator CHIR99021 (CHIR), typical VM DA neuron precursors that co-express FOXA2 and LMX1a are generated [70]. With this method, more than 70 % of the cells can be committed to a floor plate cell fate [74, 75]. The neuroepithelial cells and floor plate precursors can be induced to a VM DA neuronal fate by combination of the induction factors SHH and FGF8.

The in-vitro properties of the generated DA neurons were shown to vary between the different differentiation methods. All differentiated DA neurons were reported to be TH+ and to co-express major VM DA neuron markers NURR1, GIRK2, PITX3 etc. [73, 75, 76]; dopamine release and slow autonomous pace-making spontaneous synaptic activity was also reported [68, 77]. However, in contrast to the floor plate induced protocol [78, 79], both the embryonic body (EB) method and the stromal cell co-culture method, failed to induce FOXA2 expression during neuroepithelial cell-differentiation [47]. This later developed protocol was designed to firstly direct pluripotent cells towards floor plate cells before induction of VM DA neurons which is more similar to the in-vivo VM DA neuron development [80]. It seems the floor plate-derived DA neurons are more authentic to primary VM DA neurons [47]. Apart from more precise specification and higher efficiency, direct comparison of rosette-neuroepithelial cell-derived and floor plate cell derived DA neurons post grafting also favoured floor plate-derived DA neurons [61].

Unlike transplantation studies with human foetal VM tissue, the composition of the iPS cell-derived DA neuron grafts has been well described. By the end of in-vitro differentiation, the percentage of TH+ DA neurons derived from iPS cells varied considerably, dependent on the differentiation method used [61, 75–77, 81]. In the study by Hargus et al., 5–10 % of the total number of differentiated cells were neurons positive for tyrosine hydroxylase (TH): less than 1 % of the TH+ neurons co-expressed dopamine-β-hydroxylase (DBH), indicating that the vast majority of the TH+ neurons were DA and not noradrenergic neurons [82]. Rhee et al. reported that 50–60 % of all differentiated cells were TUJ+, of which 35–45 % were TH+ [76], whereas Kikuchi and colleagues described that, with their approach, most of the differentiated cells were TUJ+, with 85 % being TH+ [83]. Other studies reported TH-expression in 18 to 54 % of FOXA2+ cells after differentiation [61, 75]. A small contamination with serotonergic, GABAergic and glutamatergic neurons and GFAP+ astrocytes were often detected [61, 76, 77]. In general, only sporadically, undifferentiated iPS cells (indicated by the expression of SSEAs, typical pluripotency cell markers) were detected after long term differentiation [77, 82] and also a few Ki67+ proliferating cells [61, 75, 82].

### Efficacy of Intrastriatally Grafted Human iPS Cell-Derived DA Neurons in PD Animal Models

#### Behavioural Improvement and DA Neuron Survival

The studies of human iPS cell-derived DA neuron transplantation in the rat PD model are summarized in Table 2. The reduction in rotation behaviour in the unilaterally lesioned 6-OHDA rats strongly depends on the number of surviving, integrating, dopamine-producing grafted neurons. Whereas between 500 and 700 surviving foetal VM graft derived DA neurons have been shown to be sufficient to completely annihilate amphetamine-induced rotation activity, as many as 29,000 surviving human iPS cell-derived DA neurons appeared to be able to induce only a 50 % reduction in drug-induced rotation 4 weeks post grafting; less than 1500 surviving human iPS cell-derived DA neurons resulted in not any significant change in rotation behaviour. Rhee et al. reported that around 27,000 surviving TH+ cells were found in the 6-OHDA-lesioned rats that exhibited a 47.5 % reduction in rotations 8 weeks post grafting [76]. In the study by Hargus et al., the survival and integration of around 5000 human iPS cell-derived DA neurons led to a reduction in rotation of only 56 % 16 weeks post grafting [82]; these rats still did not show any improvement in the cylinder test and the adjustment stepping test [82]. The same study showed that rats containing only 350 surviving iPS cell-derived TH+ cells did not show any functional improvement in apomorphine-induced rotation [82]. Similarly, the survival of around 6700 human iPS cell-derived DA neurons reduced rotation behaviour to 78 % 16 weeks post grafting in a study by Doi et al. [75]. Kriks et al. showed that 15,000 surviving human iPS cell-derived TH+ DA neurons could fully compensate or even reverse amphetamine-induced rotation and improved the performance in cylinder test and stepping test 20 weeks post grafting [61]. In general, a much greater number of surviving TH+ DA neurons derived from human iPS cells is needed to give rise to significant functional improvement in comparison to human foetal DA neurons.

**Table 2:** Summary of grafting of human iPSC-derived DA neuron differentiated culture into PD rat model

[Ref], source (protocol: stage of graft)/ graft site	Total cell number, [cell composition]	Functional recovery	Survival of grafts (W/M post grafting)	Characteristics of grafts of DA neuron differentiated culture	Neurite outgrowth & striatum reinnervation	Side effects
[61], hiPSC lines 2C6, SeV6 (**(1)** floor plate: d25 (12 days post withdrawal of CHIR); **(2)** MS5: 14 days post withdrawal of SHH & FGF8)/ CPu	2.5 × 10^5^ cells [(1): 18 % TH+/FOXA2+, 40 % NUUR1+/FOXA2+, 95 % LMX1A+/FOXA2+ (2): 30 % TH+, 20 % Nurr1+, low in FOXA2, LMX1A]	(4.5 MPG) **Amphetamine:** (1): >100 % reduction **Stepping test**: (1): 30 % increase **Cylinder test**: (1): >10 % reduction (2): all tests n.s.	(4.5 MPG) (1): 15,000 TH+/FOXA2+ cells; serotonergic and GABA-ergic neurons <1 % (2): Few TH+ neurons	n.d.	TH+/NCAM+ fibres extended into striatum.	(2): Neural overgrowth in NOD-SCID IL2Rgc null mice
[75], hiPSC line 836B3 (floor plate, **(1)** d28, **(2)** d42 (withdrawal of purmorphamine, FGF8 at d7; CHIR99021 at day 12))/CPu	4 × 10^5^ cells (CORIN+ sorting at d12, or not) [(1): 70–75 % FOXA2+, 27.3 % NURR1+ cells, 2.1 % TH+ cells (2): 70–75 % FOXA2+, 19.9 % NURR1+ cells, 42.0 % TH+ cells]	(16 WPG) **Amphetamine:** (1): 78 % reduction (2): n.s.	(1) Sorted: 6747 TH+ cells; 2.5 % serotonin+ (1) Unsorted 3,4364 TH+ cells; 1.2 % serotonin+ (2) Sorted: 1900 TH+ cells (2) Unsorted 2000 TH+ cells.	Co-expression of TH, FOXA2, NURR1 and PITX3; 50 % of the TH+ cells co-expressed GIRK2.	More neurite outgrowth in the grafts derived from the day 12 CORIN+ sorted cells	Large graft size of unsorted grafts
[76], Lenti, retro, protein hiPSCs, hESCs (MS5: **(1)** NPCs P1-P2; **(2)** NPCs P4; **(3)** day 5 post withdraw of SHH & FGF8)/CPu	**(1)**: 7.5 × 10^5^ cells **(2)**: 3 × 10^5^ cells **(3)**: 3 × 10^5^ cells [(3): 5 % GFAP+, 60–70%TUJ1+, 35–45 % TH+ in TUJ1+ cells, 5–10 % serotonin+, a few GABAergic and glutamatergic neuronal cells]	(8 WPG) **Amphetamine** (1): 76.43 % reduction (2): 47.54 % reduction (3): 0 % reduction	(1) 54,418 TH+ cells (half size of striatum) (2) 26,882 TH+ cells (3) No TH+ cells	Co-expression of TH, TuJ1 and VMAT2; Nurr1 and EN1 Oct4+ in some lentivirus-based hiPSCs-derived neurons	n.d.	Tumor formation
[82], PDiPSC (MS5: day 42, 5 days post withdrawal of SHH & FGF8)/CPu	**(1)**: 4 × 10^5^ unsorted cells **(2)**: 4 × 10^5^ NCAM+ sorted cells 5–10 % TH+ cells	(16WPG) **Amphetamine:** (1) 56 % reduction (2) 52 % reduction **Apomorphine:** (1) 37 % reduction (2) 0 %	(1) 4890 TH+ cells (2) 350 TH+ cells	(12WPG) 53.4 % DA neurons GIRK2+; 6.6 % DA neurons calbindin+ 4.7 % forebrain DA neurons 5.8 % hypothalamic DA neurons 0.09 ± 0.02 % Ki-67+ 83.1 ± 8.2 % hNCAM+ No SSEA-4- nor Oct4+; no ubiquitin + and α-synuclein + inclusion bodies	A few TH+ fibers extended towards the striatum	n.d.

*Abbreviations: CPu* Caudate-putamen, *WPG* Weeks post grafting, *MPG* Months post grafting, *n.s.* No significant improvement, *hiPSC* Human iPSC, *PDiPSC* PD patient iPSC, *n.d.* Not determined/not described

In most studies, between 200,000 and 400,000 human iPS cell-derived cells were stereotactically transplanted in the denervated striatum of the unilaterally 6-OHDA lesioned rats. Cells were transplanted mainly at two stages of differentiation. Transplantations executed with cells at the late stage of terminal differentiation resulted in a highly variable percentages of surviving DA neurons: from 0 % [76], to only 1.1 % [75], and 5–10 % [82]. In some studies, human iPS cell-derived cells were transplanted at an early stage of dopaminergic differentiation, containing predominantly neural precursor cells and only a very few TH+ cells [75]. Such grafted neural precursor cells, already destined towards a VM DA neuron fate, could still proliferate before final differentiation into mature DA neuron in-situ and gave rise to a much larger (yet unpredictable) number of TH+ cells: 6700 [75], 15,000 [61] and 27,000 [76] TH+ cells were found within grafts. The graft size in these studies was usually much bigger than that of terminal differentiation stage implantations [75, 76]. The continuing proliferation of the neural precursor cells could result in overgrowth [76], and the early stage of differentiation could lead to the presence of undifferentiated iPS cells and so teratoma formation.

#### Characteristics of Transplanted Human iPS Cell-Derived DA Neurons

Analysis of the human iPS cell-derived grafts in the denervated striatum of the 6-OHDA lesion rats revealed TH+ DA neurons that were also positive for specific VM DA neuron markers, such as EN1, VMAT2, DAT [76] NURR1 [76], GIRK2, PITX3 [75, 83], and FOXA2 [61]. A detailed characterization of surviving human iPS cell-derived DA neurons showed that 53.4 ± 6.6 % of the TH+ cells were GIRK2+ VM DA neurons, 4.7 ± 1.0 % were GABA+ forebrain DA neurons, and 5.8 ± 1.2 % were NKX2.1+ hypothalamic DA neurons [82]. However, it should be noted that in most studies the TH+ DA neurons were actually only a small portion of the surviving human iPS cell-derived cells. The majority of the iPS cell-derived neurons appeared to be non-DA [76, 77, 82]; some were identified as serotonergic, GABAergic neurons [61, 76, 77]. GFAP+ astrocytes were also found within the grafts [76, 82]. Oligodendrocytes were never found, probably due to their very specific late generation during embryonic development and the consequently long specific in-vitro differentiation procedure required [76]. Ki-67+ proliferating cells could still be found in the intrastriatal graft [82]. Contamination of these cells may lead to graft overgrowth [76] or teratoma formation. When dopaminergic neural precursor cells were enriched by sorting for NCAM [69] or CORIN [75], graft overgrowth and teratoma formation were significantly reduced. No ubiquitin-positive and/or α-synuclein–positive inclusion bodies could be detected in grafted iPS cell-derived DA and non-DA neurons generated from fibroblasts of PD patients up to 12 weeks post grafting [82]. Remarkably, exogenous pluripotent gene (*OCT4*, *NANOG* and *SOX2*) expression persisted in subpopulations of TUJ1+ neuronal cells and TH+ DA neurons derived from lentivirally reprogrammed hiPS cells even after terminal differentiation [76]. In the study described by Hargus et al. engrafted human iPS cell-derived DA neurons showed intense arborisation and branching, yet most of the DA neurons sent TH+ fibres only towards other cells within the grafts and did not extend out to the surrounding parenchyma [82]. Kriks et al. reported that the graft core was surrounded by a halo of TH+ fibres, with some fibres occasionally extending up to 3 mm from the graft [61]. Non-TH+ neurites were found to project to their target areas according to their intrinsic phenotypic determination in specific and reproducible patterns [82]. In acute organotypic slices prepared from grafted rats, all transplanted neurons recorded showed spontaneous action potential currents.

### Human iPS Cell-Derived DA Neurons for Clinical Studies

Based on the animal studies on cell replacement therapy for Parkinson’s disease, there is no doubt that iPS cells are a prominent source for transplantable DA neurons, but the procedure to generate VM DA neurons from iPS cells in-vitro is as yet not acceptable for clinical application. A reliable quality check for human iPS cell-derived DA neurons is yet to be established. iPSC lines vary from one another both in pluripotency and differentiation efficiency [84], which may also dependent on passage number [85]. Although iPS cells resemble ESCs, a comparative study showed that some iPSC lines have a delayed formation of EBs in comparison to ESCs [85]. Different reprogramming methods may also affect the quality of iPS cell-derived DA neurons [86]. Rhee et al. found that exogenous pluripotent genes were not completely silenced at the end of the differentiation procedure. OCT4, as well as NANOG, was still detectable in subpopulations of TUJ1+ neuronal cells and TH+ DA neurons derived from lentivirally reprogrammed hiPS cells, but not in those derived from retrovirally reprogrammed hiPS cells or protein-transfer reprogrammed hiPS cells [76]. Furthermore, unlike those from protein-transfer reprogrammed hiPS cells, NP cells derived from virally-reprogrammed hiPS cells exhibited early senescence and apoptotic cell death during passaging, which was preceded by abrupt induction of p53 [76]. The variation in iPSC lines may affect the efficiency in both DA neuron differentiation and their survival post grafting.

General standards for human iPS cell-derived functional and authentic VM DA neurons are yet to be established. It is apparent that the much higher number of human iPS cell-derived DA neurons to achieve the same degree of functional improvement as that of human foetal VM DA neurons is related to the quality of the TH+ cells derived from human iPS cells. The (as yet not generally standardized) validation of human iPS cell-derived VM DA neurons is based on the expression of (a combination of) specific VM DA neuron markers, such as the co-expression of TH, LMX1A and FOXA2 [61], the co-expression of TH and GIRK2 [81], the co-expression of TH and EN1, NURR1 and PITX3, the lack of dopamine beta-hydroxylase (DBH) co-expression with TH+ cells [82], etc. In a few cases, the release of dopamine and the electrophysiology of iPS cell-derived DA neurons were used to confirm the resemblance with primary VM DA neurons [61, 76, 87]. Contamination of other cell types within the iPS cell-derived culture is a major concern, in that respect similar to the human foetal VM cell grafts. The establishment of a VM DA neuron purification method will be a breakthrough for cell replacement therapy for PD, for it will eliminate the presence of proliferating cells and undifferentiated iPS cells and reduce the risk of graft overgrowth or tumor formation. By reducing the presence of other cell types, potential cellular interactions and unknown effects of other neuronal cell types on (rotation) behaviour will be avoided. PSA-NCAM, a surface marker for neural stem/precursor cells, has been used with FAC-sorting to enrich for neural precursors [88] and floor plate-specific cell surface marker CORIN has been used to enrich for hiPS cell-derived dopaminergic neuronal precursors [75]. FAC-sorting on NCAM+/CD29^low^ cells was used by Sundberg et al. to enrich VM DA neurons [69]. Neither of these cell sorting methods resulted in a pure population of mature VM DA neurons. An alternative approach could be to generate reporter lines for DA neuronal differentiation and use reporter expression for purification or selection via FAC-sorting of human dopaminergic neurons by clinically acceptable gene modification methods [89]. It is important to note that FAC-sorting at the late stage of differentiation may hamper the viability of cells post grafting. The number of surviving DA neurons was reduced by more than 10 times in the study by Hargus et al. [82].

## Comparison of Human Foetal With Human iPS Cell-Derived Dopaminergic Neuron Grafts

Transplantation of human foetal VM tissue in PD animal models has provided proof-of-principle for DA neuron survival, outgrowth and integration as well as functional recovery of the grafted animal. It may serve as a gold standard for evaluating the quality of DA neurons derived from hiPS cells. We summarized the performance of hiPS cell-derived DA neuron grafts and compared it with that of human foetal VM tissue grafts in the 6-OHDA lesion rat model (Table 3). The number of surviving implanted TH+ neurons ranged from a few hundred to around 4000 per graft in rats grafted with human foetal VM tissue. About 500–700 of viable functional DA neurons appeared to be sufficient to reverse drug-induced rotation under optimal surgery conditions [38, 44]. On the contrary, survival of TH+ cells from iPS cell-derived dopaminergic neuron grafts ranged from a few to 29,000. In contrast to the human foetal DA grafts, thousands of iPS cell-derived TH+ cells appeared to be needed to fully reverse drug-induced rotation [61, 75].

**Table 3:** Comparison of major parameters of (non-purified) human foetal and human iPSC-derived dopaminergic grafts in rat PD model

	Human foetal DA graft	Human iPSC-derived DA graft
Composition	DA neurons, 5-HT neurons, GABAergic neurons, astrocytes, microglia [32, 34]	DA neurons, NSCs, undifferentiated iPSCs serotonergic, GABAergic neurons astrocytes [75–77, 81, 82]
Percentage DA neurons in original graft	5.6 % [32, 33]	5–85 % [61, 75–77, 81, 82]
Survival	++++ 1–10 % [35, 37]	+++ 0–12 % [75, 76, 82]
Graft overgrowth	–	++ [76]
Migration	+ [37]	not detected
Neurite outgrowth	Long extensions [34, 36, 38, 40, 44]	Short extensions [61, 82]
Synaps formation	++ [34, 38]	not detected
Recovery (rotation reduction, other tests)	++++ [35, 38, 44]	++ [75, 76]
Minimal number of TH+ cells required to full reversion of rotation behaviour	500–700 [38, 44]	15,000 [61]
Co-expression of VM DA markers in surviving DA neurons	FOXA2+, LMX1a+, EN1+, NURR1+, GIRK2+, TH+ [47, 69]	TH+, GIRK2+, EN1+, NURR1+, LMX1A+, and PITX3+ [61, 75, 76]
Intrastriatal DA release	++ [34, 37]	not tested
Suppression of striatal interneurons	+++ [40]	not tested
Characteristic DA neuron firing in-vivo	++++ [40]	not tested

**Fig. 2:**
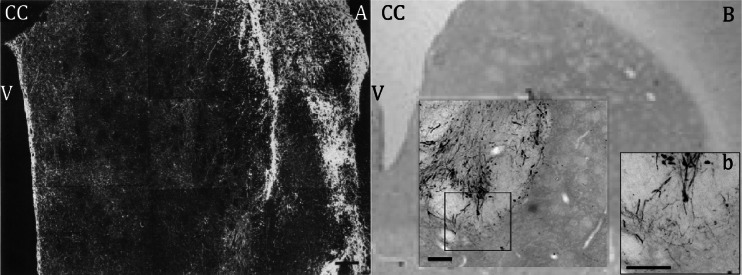
Comparison of grafted human foetal VM tissue and hiPS cell-derived DA neurons. **a** Overview of TH+ neurites in a coronal section of the striatum 4 months after grafting human foetal VM tissue from a 10-week-old foetus. The graft, filled with TH+ cell bodies and fibres is seen at the far right, TH+ processes radiate into host striatum and form a network that covers the whole area of striatum. **b** Overview of TH+ iPS cell-derived DA neurons (unsorted) 4 months after intrastriatal transplantation. Higher magnification showing TH-positive somata and some innervation of the surrounding host striatum by graft-derived neurites (*b*) Scale bar = 200 μm, *CC* = corpus callosum, *V* = ventricle. Adapted from [40, 75]

Primary DA neurons from foetal VM tissue gave rise to extensive neurite outgrowth up to 6 mm [37] and reinnervated the whole volume of the striatum (Fig. 2a) [38, 40]. Neurite outgrowth of iPS cell-derived DA neurons varies from case to case but, in general, it remained relatively limited [82] or extended only 2–3 mm into the striatum, reinnervating only around 10 % of the striatum, despite the large number of surviving TH+ neurons (Fig. 2b) [61, 75]. It seems likely that the more limited survival and neurite outgrowth of grafted hiPS cell-derived DA neurons in comparison to the primary human foetal DA neurons, is responsible for a lower degree of recovery of the 6-OHDA lesion rats in the drug-induced rotation behaviour. In contrast to hESC-derived DA neurons [90], data about dopamine release or the electrophysiological characteristics of hiPS cell-derived DA neurons integrated in the striatum after grafting are not yet available.

## Conclusions

Although the human iPS cell-derived DA neurons seem to fulfil the major criteria for functional dopamine-releasing neurons, it is clear that they were not as effective as the human primary (foetal) DA neurons in restoring drug-induced rotation behaviour in the unilaterally 6-OHDA lesion rats. A much higher number of surviving DA neurons were required to obtain the same behavioural effects of the primary foetal DA neuron grafts. The human iPS cell-derived DA neurons showed a lower survival rate after striatal implantation and their neurite outgrowth was not as extensive as that of the human primary foetal DA neurons. Apparently, the human DA neurons obtained via differentiation from iPS cells were not identical to the primary DA neurons isolated from the VM region of the aborted foetuses. Indeed, after the reprogramming-associated complete juvenation, the iPS cell-derived DA neuron may also be considered foetal, but the differentiation protocol most likely did not result in the very specific population of foetal A9 DA neurons that have the specific intrinsic properties to innervate the striatum. It is likely that, after the drastic iPS cell-reprogramming and the subsequent dopaminergic differentiation, fibroblast-derived epigenetic marks were still present interfering with a proper complete reprogramming and DA differentiation. In that respect, it is of considerable interest to note that DA neurons differentiated from human ESCs (with a similar protocol used for hiPS cells) recently showed long-term survival, efficacy in restoration of motor function as well as long extensive neurite outgrowth and wide striatal innervation after implantation in the rat PD model with a potency comparable to that seen with human foetal dopamine neurons [91]. Recently, we performed an in-depth comparative analysis of the expression profile of pure mouse iPS cell-derived DA neurons with that of pure mouse primary DA neurons [92]. We used Ptix3-GFP mice to enable the isolation of pure iPS cell-derived DA neurons and pure primary VM DA neurons with GFP-based FAC-sorting. Besides comparative global gene expression mapping, we performed comprehensive DNA methylation profiling by Reduced Representation Bisulfite Sequencing (RRBS). The mouse iPS cell-derived DA neurons indeed largely appeared to adopt characteristics of their in vivo counterparts, in morphology, dopamine production, global gene expression and CpG island (CGI) methylation profiles, though some fibroblast genes appeared to be expressed still [92]. However, we also found deviations in CGI methylation for a subpopulation of genes which clearly effected predominately the expression of genes associated with functional annotations such as “nervous system development”, “neurogenesis” and “neuron differentiation and outgrowth” [92]. It is likely that similar iPS reprogramming “artefacts” may be responsible for the limited outgrowth and survival of the human iPS cell-derived DA neurons after implantation in the rat Parkinson model. Extensive comparative expression profiling combined with genome wide epigenome analysis, like we have done for the mouse, is needed to examine in more detail the differences between human primary foetal DA neurons and the human iPS cell-derived DA neurons, although proper purification of human DA neurons may still be a bottle neck for that.

In view of the considerable undeniable clinical potential of (autologous) human iPS cell-derived DA neuron grafts, fundamental and preclinical research is still progressing which will eventually lead to a first clinical trial on a small number of Parkinson patients, presumably within 2 years. Important intermediate steps towards such trial have been taken in recent studies on the transplantation of autologous iPSC-derived dopamine neurons in non-human primate model of Parkinson’s disease [93, 94], showing the redundancy of the use of immunosuppressiva.
